# Correlation Between Serum Vitamin D and Calcium Levels and the Risk of Dental Caries in Children in Western Maharashtra, India

**DOI:** 10.7759/cureus.76340

**Published:** 2024-12-24

**Authors:** Sandisha S Sudrik, Sachin Gugawad, N. D. Shashikiran, Namrata Gaonkar, Savita G Hadakar, Sonali Waghmode

**Affiliations:** 1 Department of Paedodontics and Preventive Dentistry, School of Dental Sciences, Krishna Vishwa Vidyapeeth (Deemed to Be University), Karad, IND

**Keywords:** calcium, caries, children, maharashtra, vitamin d

## Abstract

Background

Vitamin D and calcium are necessary for tooth health, and a lack of these can cause substantial changes. Dental caries, or tooth decay, is a common childhood illness that causes pain, difficulty chewing, and a lower quality of life. There is a scarcity of research on the correlation between vitamin D and calcium levels and dental caries risk among children in western Maharashtra.

Aim and objectives

This article aims to study the correlation between vitamin D and calcium levels in serum with the risk of dental caries in children in western Maharashtra.

Methodology

The study was conducted on 124 children whose blood samples were taken to test for serum vitamin D and serum calcium levels. The DMFT (Decayed, Missing, and Filled Teeth)/DMFS (Decayed, Missing, and Filled Surfaces) scores of all subjects were recorded. Data obtained were subjected to statistical analysis.

Results

The mean age of the subjects was 10.04 ±0.91 years. A statistically significant negative linear relationship was found between serum vitamin D, calcium, and dental caries experience (DMFT and DMFS scores). DMFT and DMFS scores for dental caries decrease as blood levels of vitamin D and calcium rise. The logistic regression study indicated that serum vitamin D has a negative regression coefficient (-0.23) and an odds ratio of 0.4, demonstrating that caries risk and vitamin D levels rise in opposite directions at 60%.

Conclusion

The study shows a significant correlation between serum vitamin D levels and the risk of dental caries in children in western Maharashtra.

## Introduction

Vitamin D, a fat-soluble vitamin, plays an essential role in maintaining overall health. Its importance extends beyond general health to include vital functions in systemic health, bone health, and dental health across all stages of life. Recent research has highlighted vitamin D’s crucial role in regulating the metabolism of calcium and phosphate, two minerals that are essential for the development and maintenance of mineralized tissues, including bones and teeth. Given its multifaceted influence, vitamin D is the focus of increasing attention in both medical and dental research fields [1].

One of the primary roles of vitamin D is its regulation of calcium and phosphate metabolism. These minerals are essential for the formation and maintenance of strong bones and teeth. Calcium helps to build and maintain bone density, while phosphate is a key component of tooth enamel and bone mineralization. Adequate vitamin D levels are critical for the efficient absorption of calcium and phosphate from the intestines, ensuring that these minerals are available for proper skeletal and dental development. A deficiency in vitamin D, especially during childhood and adolescence, can lead to poor bone mineralization, enamel hypoplasia, and an increased susceptibility to dental caries (tooth decay) [2].

Dental caries is the most prevalent oral disease globally, affecting a significant proportion of children and adolescents. It is a multifactorial disease, with numerous contributing factors, including diet, oral hygiene, and genetic predisposition. Among these factors, vitamin D deficiency has been increasingly recognized as a major risk factor for the development of dental caries. Research has shown that children who experience vitamin D deficiency, particularly during the development of both primary and permanent teeth, have a higher likelihood of developing dental caries compared to their peers with sufficient vitamin D levels [3]. This association is thought to be linked to the role of vitamin D in calcium metabolism and its influence on the formation of tooth enamel.

The mechanisms by which vitamin D influences dental health are complex. One of the key physiological processes is the ability of vitamin D to enhance calcium absorption in the intestines. When calcium intake is insufficient, the parathyroid hormone (PTH) is activated. PTH not only releases calcium from the body’s skeletal reserves but also stimulates the conversion of 25-hydroxyvitamin D to its active form, 1,25-dihydroxyvitamin D3, which further facilitates calcium absorption from the intestines. This process is critical for maintaining adequate calcium levels in the body, particularly for developing teeth and bones. However, when vitamin D levels are low, this process is impaired, leading to weakened tooth enamel and an increased risk of dental decay [4].

Serum vitamin D levels are typically classified into three categories: levels below 30 nmol/L are considered deficient, levels between 30 nmol/L and 50 nmol/L are classified as insufficient, and levels above 75 nmol/L are considered optimal for maintaining good health [5]. A growing body of evidence suggests that a significant number of children and adolescents worldwide, particularly in regions with limited sunlight or in populations with darker skin tones, exhibit suboptimal vitamin D levels. Such deficiencies are more pronounced in regions where there is limited sun exposure, which is a primary source of vitamin D synthesis in the body. Individuals with darker skin pigmentation have a reduced capacity to synthesize vitamin D from sunlight, making them more susceptible to deficiency, especially in areas with less sunlight exposure [6]. Vitamin D is a significant predictor of dental caries due to its essential role in maintaining oral health through calcium and phosphate metabolism, which are critical for tooth mineralization and enamel integrity. Vitamin D deficiency increases the risk of dental caries, with a meta-analysis demonstrating a 22% higher prevalence of caries among children with low serum vitamin D levels. This relationship is attributed to vitamin D's influence on enamel formation, its role in enhancing antimicrobial peptide production, and its contribution to salivary gland function, which collectively help reduce bacterial load and acid production in the oral cavity.

Despite the well-documented role of vitamin D in bone health and systemic health, there is a relative scarcity of research exploring the relationship between serum vitamin D levels and the incidence of dental caries in specific populations. Most existing studies focus on broader health outcomes or are conducted in regions outside of India. In particular, there is limited research investigating the correlation between serum vitamin D and calcium levels and their relationship with dental caries in children residing in Maharashtra, India. This region, which experiences seasonal variation in sunlight and has diverse dietary patterns, offers a unique context in which to explore this relationship.

Therefore, the primary aim of this study is to investigate the association between serum vitamin D and calcium levels and the likelihood of dental caries among children in western Maharashtra. By filling this gap in existing research, this study seeks to provide valuable insights into the role of vitamin D in oral health, particularly in children, and to highlight potential public health interventions for reducing the burden of dental caries in this population.

## Materials and methods

Study design

This investigation employed a correlational design aimed at examining the relationship between serum vitamin D and serum calcium levels and the likelihood of dental caries in children. The study was designed to explore how these two serum levels may correlate with dental health outcomes, particularly focusing on the DMFT (Decayed, Missing, and Filled Teeth) and DMFS (Decayed, Missing, and Filled Surfaces) scores as measures of dental caries.

Study setting

The study was conducted at the Department of Paedodontics and Preventive Dentistry, School of Dental Sciences, Krishna Vishwa Vidyapeeth (Deemed to Be University), Karad. Ethical approval for the study was obtained from the Institutional Ethical Committee prior to the start of data collection. Following the acquisition of informed consent from the participants or their guardians, blood samples were collected for analysis of serum vitamin D and calcium levels. In addition to these biochemical measures, the DMFT and DMFS scores were recorded for all subjects to assess the extent of dental caries, which formed the primary health outcomes of the study.

Sample size

The sample size was determined based on the results of a pilot study that included 20 participants. The pilot study yielded a correlation coefficient of 0.28, with a statistical power of 90% and a margin of error of 5%. These parameters led to the calculation of a required sample size of 124 participants. To ensure sufficient statistical power, the sample size was rounded up to 130 participants. Participants were selected through a simple random sampling procedure, ensuring that each participant had an equal chance of being chosen, which minimized selection bias.

Inclusion and exclusion criteria

The study included children aged between 7 and 13 years who were in the mixed dentition phase - a stage where both primary and permanent teeth are present. The cohort included both males and females from various socio-economic backgrounds. Children with a history of dental caries - meaning they had decayed, missing, or filled teeth or surfaces - were included in the study. On the other hand, children with any systemic disease that could interfere with serum vitamin D or calcium levels, or those whose dental health could be influenced by such conditions, were excluded. This was done to ensure that the study focused specifically on the relationship between serum levels and dental caries, without the confounding effect of underlying medical conditions.

Study parameters

The study focused on several key parameters to assess both the biochemical and dental health status of the participants. Serum vitamin D levels were measured in nanograms per milliliter (ng/mL), and serum calcium levels were measured in milligrams per deciliter (mg/dL). Basic demographic data, including age and gender, were collected for each participant to account for potential confounding variables. The dental health outcomes were assessed using the DMFT and DMFS scores, which provided an indication of the extent of dental caries based on the number of decayed, missing, or filled teeth and surfaces. These parameters were critical for understanding how the two serum levels correlated with dental health.

Statistical analysis

The data collected from the study participants were analyzed using a combination of descriptive statistics and inferential statistical methods. Continuous data, such as serum vitamin D and calcium levels, were summarized using mean and standard deviation, while categorical data, such as gender and age group, were summarized using frequency and percentage. To assess the relationship between serum calcium and vitamin D levels and the DMFT and DMFS scores, Pearson's correlation coefficient was employed to measure the strength and direction of the linear relationships. Furthermore, a multiple logistic regression analysis was performed to explore the association between serum vitamin D and calcium levels and the odds of having dental caries, as measured by the DMFT and DMFS scores. A significance level of p < 0.05 was set for all statistical tests, ensuring that only statistically significant results were considered in the interpretation.

## Results

This study analyzed a cohort of participants with an average age of 10.04 ± 0.91 years. Demographic characteristics of the sample revealed a higher proportion of female participants compared to males in both groups. This gender distribution provided a diverse foundation for examining the relationship between biochemical markers and dental caries experience (Table 1). The analysis demonstrated a statistically significant negative linear correlation between serum vitamin D levels, serum calcium levels, and dental caries experience, as measured by DMFT and DMFS scores. Specifically, as serum concentrations of vitamin D and calcium increased, there was a notable reduction in the DMFT and DMFS scores. This finding suggests an inverse relationship, wherein higher levels of these biochemical markers are associated with improved dental health outcomes (Table 2). These results highlight the potential protective role of vitamin D and calcium against the progression of dental caries.

**Table 1:** Descriptive statistics The data provides a detailed summary of the demographic characteristics and key health parameters of the study participants. Out of a total sample of 130 individuals, 63 participants (48.46%) are male, and 67 participants (51.54%) are female, indicating a nearly equal gender distribution. The age distribution ranges from 8 to 12 years, with the highest proportion of participants being 11 years old (31 participants, 23.85%), followed by 12 years old (29 participants, 22.31%), eight years old (n26 participants, 20.00%), nine years old (23 participants, 17.69%), and 10 years old (21 participants, 16.15%). In terms of key health parameters, the average vitamin D level is 25.25 ng/mL with a standard deviation (SD) of 8.05, while the mean serum calcium level is 9.75 mg/dL with an SD of 0.78. The mean DMFT score (Decayed, Missing, and Filled Teeth) is 3.26 with an SD of 1.51, and the average DMFS score (Decayed, Missing, and Filled Surfaces) is 3.81 with an SD of 1.50.

		N	Percentage (%)
Sex	Male	63	48.46
	Female	67	51.54
Age	8	26	20.00
	9	23	17.69
	10	21	16.15
	11	31	23.85
	12	29	22.31
		Mean	SD
Vitamin D	-	-25.25	8.05
Serum Calcium	-	9.75	0.78
DMFT	-	3.26	1.51
DMFS	-	3.81	1.5

**Table 2:** Correlation of serum vitamin D and calcium levels with DMFT and DMFS scores DMFT: Decayed, Missing, and Filled Teeth; DMFS: Decayed, Missing, and Filled Surfaces *Statistical significance set at 0.05; r: Pearson’s correlation coefficient The data reveals the correlation between vitamin D levels and serum calcium levels with dental health indicators (DMFT and DMFS scores). A significant negative correlation is observed between vitamin D levels and both DMFT and DMFS scores. Specifically, the correlation coefficient (r) for vitamin D and DMFT is -0.74 (P = 0.001), and for vitamin D and DMFS, it is -0.72 (P = 0.001). This indicates that higher vitamin D levels are strongly associated with lower DMFT and DMFS scores, suggesting better oral health (fewer decayed, missing, and filled teeth/surfaces) with increased vitamin D. For serum calcium levels, a weaker but still significant negative correlation is observed. The correlation coefficient (r) for calcium and DMFT is -0.19 (P = 0.033), and for calcium and DMFS, it is -0.28 (P = 0.01). This suggests that higher serum calcium levels are also associated with lower DMFT and DMFS scores, but the relationship is less pronounced compared to vitamin D. Overall, these results highlight the important role of vitamin D in oral health, as its strong negative correlation with DMFT and DMFS scores implies a protective effect against dental caries and tooth surface damage. While calcium levels also show a negative correlation, its effect appears to be moderate. The statistically significant P-values (all below 0.05) confirm the reliability of these findings.

	DMFT		DMFS	
	r	P-value	r	P-value
Vitamin D test	-0.74	0.001*	-0.72	0.001*
Calcium test	-0.19	0.033*	-0.28	0.01*

For instance, participants with lower serum vitamin D and calcium levels consistently exhibited higher DMFT and DMFS scores, reflecting a greater burden of dental caries. Conversely, individuals with elevated levels of these nutrients tended to have fewer decayed, missing, and filled teeth or surfaces, underscoring the importance of adequate nutritional and biochemical status in maintaining oral health. To further investigate the influence of serum vitamin D and calcium on dental caries risk, a multiple logistic regression analysis was conducted. The dependent variable in this model was the DMFS score categorized into "High risk," while the predictors included serum vitamin D and calcium levels. The regression model as a whole was statistically significant (Chi²(4) = 77.16, p < 0.001, n = 130), indicating that the predictors collectively explained a substantial proportion of the variability in dental caries risk (Table 3).

**Table 3:** Association of serum vitamin D, serum calcium levels in predicting the caries risk *Statistical significance set at 0.05; Model of prediction: High-risk caries (Decayed, Missing, and Filled Surfaces) The data presents the relationship between serum vitamin D and serum calcium levels with dental health outcomes using odds ratios (OR) and P-values. For serum vitamin D, the coefficient is -0.23, with an OR of 0.8 and a P-value of <0.001. This indicates a statistically significant negative association between vitamin D levels and the likelihood of dental issues. An OR of 0.8 means that for every unit increase in serum vitamin D, the odds of having dental caries (or related outcomes) decrease by approximately 20% (1-0.8 = 0.2 or 20%). The P-value (< 0.001) confirms that this result is highly significant and unlikely to be due to chance, highlighting the protective role of vitamin D in dental health. For serum calcium, the coefficient is 0.63, with an OR of 1.87 and a P-value of 0.118. This indicates a positive but non-significant association between serum calcium levels and the odds of dental issues. An OR of 1.87 suggests that higher calcium levels might increase the odds of dental issues by 87%, but the P-value (0.118) indicates that this result is not statistically.

	Coefficient (Odd’s Ratio)	P-value
Serum Vitamin D	-0.23 (0.8)	<0.001*
Serum Calcium	0.63 (1.87)	0.118

**Figure 1:**
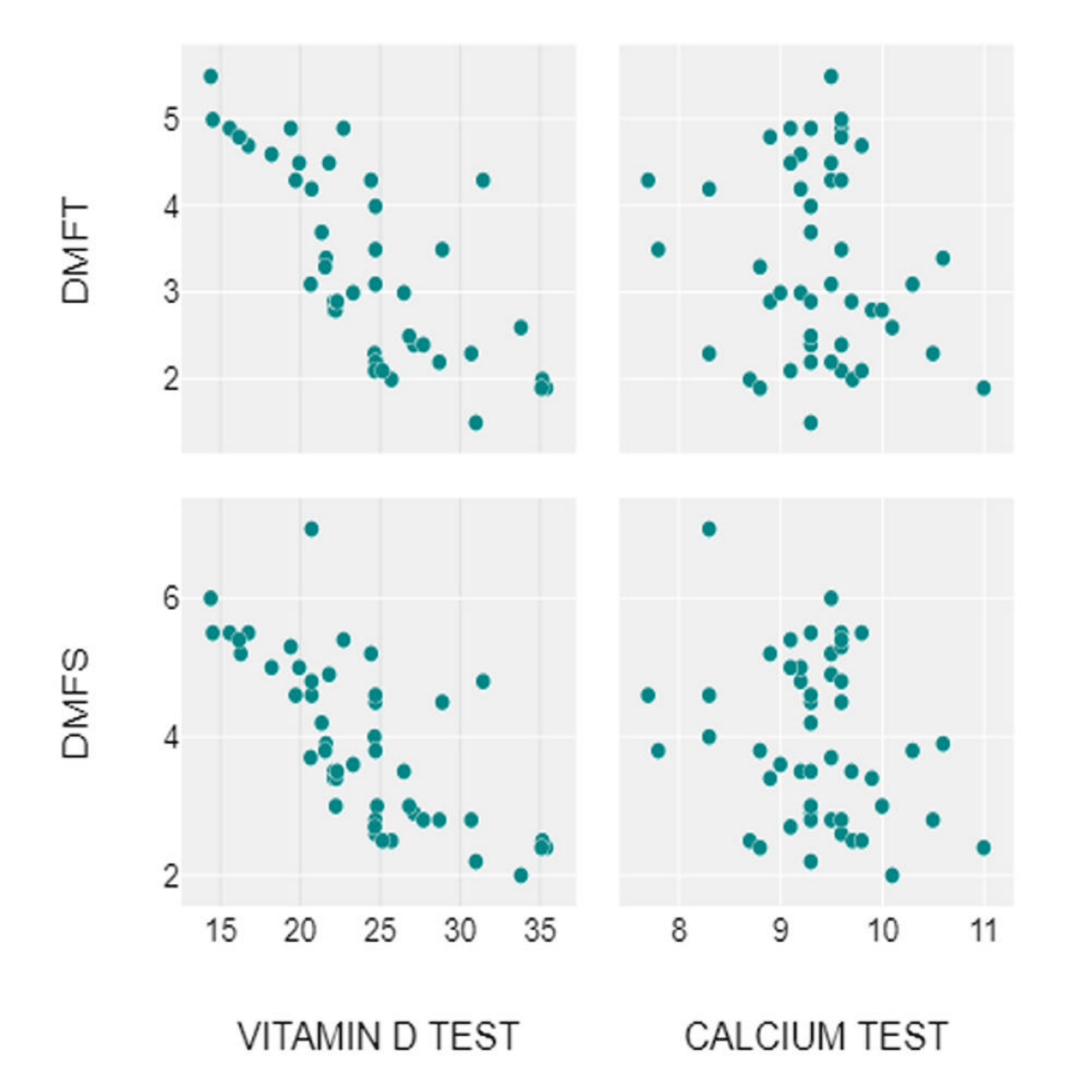
Scattered diagram showing distribution of serum vitamin D and calcium levels with DMFT and DMFS scores DMFT: Decayed, Missing, and Filled Teeth; DMFS: Decayed, Missing, and Filled Surfaces The scatter plots demonstrate the relationships between serum vitamin D and calcium levels with DMFT and DMFS scores. In the vitamin D test vs. DMFT plot (Top-Left), there is a noticeable negative correlation, indicating that as vitamin D levels increase, DMFT scores tend to decrease. This suggests that higher vitamin D levels might be associated with better dental health, as fewer teeth are affected by decay, missing, or fillings. Similarly, the vitamin D test vs. DMFS plot (Bottom-Left) shows a comparable negative correlation, where higher vitamin D levels are associated with lower DMFS scores. This reinforces the potential protective role of vitamin D in maintaining healthier teeth and reducing decay on tooth surfaces. On the other hand, the relationship between calcium levels and DMFT scores (Top-Right) appears scattered, with no clear upward or downward trend, indicating no significant correlation. This suggests that calcium levels do not strongly influence the number of decayed, missing, or filled teeth. A similar pattern is observed in the calcium test vs. DMFS plot (Bottom-Right), where the points remain scattered without a clear trend, showing no noticeable correlation between calcium levels and tooth surface health.

The combined findings from the correlation and regression analyses strongly support the hypothesis that higher serum levels of vitamin D and calcium are protective against dental caries. Vitamin D plays a pivotal role in calcium absorption and utilization, and together these nutrients contribute to maintaining the structural integrity of teeth and enhancing resistance to decay. The observed reduction in DMFT and DMFS scores with increasing serum levels of these markers suggests that nutritional and biochemical interventions could be effective strategies for caries prevention (Figure 1).

Moreover, the logistic regression results provide valuable insights for risk prediction. By identifying individuals with low serum vitamin D and calcium levels as being at heightened risk for severe dental caries, targeted interventions can be designed to address these deficiencies. These findings emphasize the need for comprehensive public health strategies that integrate nutritional education and supplementation to promote optimal oral health, particularly in populations with a high prevalence of vitamin D or calcium deficiencies. Overall, the results of this study highlight the intricate relationship between systemic biochemical markers and localized dental outcomes, providing a compelling argument for the inclusion of nutritional assessments in routine dental care practices.

## Discussion

This study aimed to investigate the correlation between serum levels of vitamin D and calcium with the risk of dental caries in children residing in western Maharashtra. The results revealed a strong negative association between serum vitamin D levels and the likelihood of dental caries, which is consistent with previous research. Studies have shown that children with lower vitamin D levels are more likely to experience dental caries than those with adequate levels of vitamin D [7-9]. This suggests that vitamin D plays a critical role in oral health, particularly in maintaining enamel integrity and preventing decay.

Vitamin D is a vital nutrient for the development and maintenance of healthy teeth and bones. It is involved in the absorption of calcium and phosphorus, both of which are essential for tooth mineralization. The current study supports the growing body of evidence indicating that children with low serum vitamin D levels are more prone to dental caries. This finding aligns with the results of Schroth et al. (2013), who found that children with insufficient vitamin D were significantly more likely to develop caries compared to those with adequate levels [8]. Additionally, our study observed that children with lower vitamin D levels had higher caries incidence, reinforcing the idea that vitamin D is a crucial factor in oral health.

Several mechanisms have been proposed to explain the association between vitamin D deficiency and dental caries. Vitamin D is known to activate antimicrobial proteins such as cathelicidins and defensins, which help maintain oral health by reducing bacterial colonization on teeth and gums [10,11]. A deficiency in vitamin D leads to a reduction in the expression of these proteins, which may contribute to an increased risk of dental infections and caries. Furthermore, vitamin D plays a vital role in maintaining adequate salivary flow, which helps neutralize acids in the mouth and prevents demineralization of tooth enamel. Studies have shown that vitamin D deficiency leads to reduced salivary flow and a decrease in the calcium content of saliva, both of which are important in preventing dental caries [12].

Vitamin D deficiency is associated with enamel hypoplasia - a condition that weakens tooth enamel and makes it more susceptible to decay. Vitamin D supplementation could be an effective strategy to improve oral health, especially in children who are at a higher risk for dental caries due to vitamin D deficiency [13]. Previous studies have suggested that calcium plays an important role in protecting tooth enamel and reducing caries risk [14]. For example, Karshan (1939) observed that lower calcium concentrations in saliva were associated with increased caries activity, suggesting a protective role for calcium in maintaining enamel integrity [15]. However, the results of our study did not support this association, which could be due to several factors.

The lack of a significant relationship between calcium levels and caries risk may be due to the complex nature of calcium metabolism, which is influenced by various factors, including dietary intake, hormonal regulation, and the levels of vitamin D. Calcium absorption is significantly affected by vitamin D, and a deficiency in vitamin D can impair calcium absorption, thus potentially masking any effect of calcium on caries risk. Moreover, the bioavailability of calcium in the oral cavity may vary depending on its interaction with other nutrients and the presence of salivary calcium-binding proteins. Therefore, the role of calcium in preventing dental caries may be influenced by the presence of other factors, such as vitamin D levels.

The physiological role of PTH in calcium homeostasis may provide insights into the observed lack of association between serum calcium levels and caries risk in this study. PTH is a critical regulator of serum calcium levels, acting to maintain these levels within a narrow range by stimulating osteoclastic activity, enhancing bone resorption, and releasing calcium into the bloodstream during periods of calcium deficiency. This mechanism ensures systemic calcium stability, even in cases of dietary insufficiency or other disruptions. However, this regulatory process may obscure any potential association between systemic calcium levels and dental caries risk. While serum calcium remains tightly regulated, local calcium availability in the oral cavity, particularly in saliva, plays a more direct role in enamel remineralization and caries prevention. Salivary calcium concentrations are influenced by dietary calcium intake, salivary gland function, and other local factors rather than directly reflecting systemic calcium levels.

Moreover, dental caries is a multifactorial disease influenced by variables such as dietary sugar consumption, oral hygiene practices, microbial biofilm activity, pH levels, and fluoride exposure. These local factors likely exert a more significant impact on caries development than systemic calcium levels. The role of PTH in bone metabolism, while crucial for calcium homeostasis, may also have indirect implications, such as affecting jawbone structure or tooth stability, but these are not directly tied to the etiological mechanisms of caries, which are primarily driven by local oral environmental factors.

Furthermore, it is possible that the influence of calcium on dental caries is more complex and may depend on other variables such as dietary intake and oral hygiene habits, which were not assessed in this study. Therefore, further research is needed to clarify the relationship between calcium and caries risk. Our findings regarding vitamin D are consistent with several studies that have established a significant association between low vitamin D levels and an increased risk of dental caries. For example, Schroth et al. (2013) found that children with inadequate vitamin D levels had a much higher risk of dental caries compared to those with sufficient levels of vitamin D [14]. However, the lack of a significant correlation between calcium levels and dental caries in our study differs from the findings of other studies, such as Karshan (1939), who suggested that calcium levels play a protective role against caries by maintaining enamel integrity [15].

Recent studies have focused on the potential role of vitamin D in oral health, particularly its effect on the prevention of dental caries. Li et al. (2023) conducted a meta-analysis specifically looking at the correlation between serum vitamin D levels and the risk of dental caries in children. The study reinforced the idea that vitamin D plays a protective role, with higher vitamin D levels being inversely associated with the risk of developing caries [16]. The study suggests that vitamin D is an important nutrient for maintaining oral health, particularly in preventing dental caries in both children and adults. Ensuring sufficient vitamin D intake may be a cost-effective strategy to reduce the burden of dental diseases globally. These discrepancies may be attributed to differences in study populations, methodologies, and sample sizes, as well as the potential confounding factors not addressed in our study.

Maternal vitamin D deficiency during pregnancy plays a crucial role in the oral health of the child, particularly in the mineralization of developing teeth. Vitamin D is essential for the proper absorption of calcium and phosphorus, which are critical for the formation of hard tissues such as bones and teeth. Insufficient vitamin D levels in the mother during pregnancy can result in fetal vitamin D deficiency, which impairs the mineralization of primary and permanent teeth. This condition often leads to enamel hypoplasia, a developmental defect characterized by thin or poorly mineralized enamel, which significantly increases the susceptibility of teeth to dental caries.

These findings underline the importance of ensuring adequate maternal vitamin D levels during pregnancy for proper dental development in children. Public health measures aimed at monitoring and supplementing vitamin D levels in pregnant women could play a vital role in reducing the risk of dental caries in their offspring. A preventive or intervention protocol for patients with decreased serum vitamin D should focus on early detection, dietary adjustments, and supplementation. Regular screening for vitamin D deficiency, especially in children with a history of dental caries or delayed tooth development, is essential for timely intervention. Based on previous studies, dietary recommendations should include foods rich in vitamin D, such as fortified milk, eggs, and fatty fish. When dietary intake is insufficient, vitamin D supplementation (typically 400-1000 IU/day) can be recommended to improve serum levels and support proper calcium absorption, which is crucial for tooth mineralization. Ensuring adequate calcium intake through dietary sources like dairy products and leafy greens is equally important, as vitamin D enhances calcium absorption. Regular follow-up visits to monitor serum vitamin D levels and adjust the protocol as needed are also recommended. In addition, educating parents about the importance of vitamin D and calcium for their child’s oral health, along with promoting good oral hygiene practices and regular dental check-ups, can further reduce the risk of dental caries. Further research into maternal and child nutrition and its effects on oral health may provide deeper insights into preventive strategies.

The limitations of this study include several important factors that should be considered. While it provides valuable insights into the relationship between serum vitamin D and calcium levels and dental caries, there are certain constraints. First, the study's cross-sectional design limits our ability to establish causality. While we observed an association between low vitamin D levels and increased caries risk, further longitudinal studies are required to determine whether vitamin D deficiency directly causes dental caries or if other factors contribute to this relationship over time.

Another limitation is the lack of dietary data, which could have provided a better understanding of the factors influencing vitamin D and calcium levels. The study did not account for sun exposure, an important factor for vitamin D synthesis, nor did it assess dietary intake of vitamin D or calcium, both of which can significantly affect serum levels of these nutrients. Furthermore, ethnicity was not considered, which could influence the findings due to differences in vitamin D metabolism across ethnic groups. Additionally, the study did not consider the oral hygiene practices or fluoride exposure of participants, both of which are important factors in preventing dental caries. Finally, the study did not assess other potential confounding factors, such as socioeconomic status, which could affect access to dental care, nutrition, and overall oral health.

## Conclusions

This study is significant for pediatric dentists as it emphasizes the crucial role of vitamin D and calcium in preventing dental caries. By understanding these relationships, pediatric dentists can better implement preventive strategies, monitor potential nutritional deficiencies, and provide timely interventions to reduce caries risk. The study also promotes a holistic approach to patient care by recognizing the broader impact of overall health on oral health. Furthermore, it supports evidence-based practices, enabling pediatric dentists to offer more informed and effective treatments and guidance to improve children's oral health outcomes.

In conclusion, the study highlights the importance of vitamin D in preventing dental caries in children, with lower levels of vitamin D being linked to an increased risk of caries. While no significant association was found between calcium levels and caries risk, further research is necessary to explore the complex relationship between these nutrients and oral health. The study also calls for future investigations that account for factors like dietary intake, sun exposure, and other potential confounders, as well as longitudinal studies to establish causal links. Vitamin D supplementation could be considered as a preventive measure, especially for children with low vitamin D levels, to enhance oral health.
